# Genome-wide analysis of MpBHLH12, a IIIf basic helix-loop-helix transcription factor of *Marchantia polymorpha*

**DOI:** 10.1007/s10265-019-01095-w

**Published:** 2019-03-06

**Authors:** Haruka Arai, Kazuya Yanagiura, Yuko Toyama, Kengo Morohashi

**Affiliations:** Department of Applied Biological Science, Faculty of Science and Technology, Tokyo University of Science, 2641, Yamazaki, Noda, Chiba 278-8510 Japan

**Keywords:** *Arabidopsis thaliana*, bHLH transcription factor, Gene regulatory network, *Marchantia polymorpha*

## Abstract

The evolution of plants on land required adaptation to UV radiation and dry environments, and involved the appearance and/or rewiring of genetic connections, known as gene regulatory networks (GRNs), which consist of one or more transcription factors (TFs). The liverwort, *Marchantia polymorpha*, is a basal land plant, with a recently sequenced genome. The number of genes encoding basic helix-loop-helix (bHLH) family members is considerably higher in *M. polymorpha* than in charophyte green algae, suggesting the contribution of bHLH proteins to the evolution of GRNs associated with the adaptation of plants to land. Although an understanding of the evolutionary aspects of GRNs is fundamental for elucidating the mechanisms of environmental adaptation, the evolution of GRNs that led to land adaptation in plants remains poorly understood. In this study, we isolated a single gene encoding a IIIf bHLH TF from *M. polymorpha, MpBHLH12*. Transgenic *M. polymorpha* constitutively overexpressing *MpBHLH12* showed smaller and fewer gemma cups than wild type, suggesting that MpBHLH12 is involved in the regulation of morphological development. Transcriptomic analysis of *MpBHLH12* overexpressor (MpBHLH12ox) lines revealed an overlap with the GRN of *MpMYB14*, which regulates the biosynthesis of anthocyanins and phenolic compounds. However, MpBHLH12ox did not show anthocyanin accumulation. Results of the transient reporter assay suggest that *MpBHLH12* could function in repression rather than activation. Our findings suggest that although the IIIf bHLH MpBHLH12 shows highest amino acid similarity with IIIf bHLH clade and is involved in developmental process and partly biosynthesis of phenolic compounds in *M. polymorph*a like Arabidopsis IIIf bHLH, the GRN involving *MpBHLH12* would be distinct one from those of the IIIf bHLH TFs of seed plants.

**Electronic supplementary material:**

The online version of this article (10.1007/s10265-019-01095-w) contains supplementary material, which is available to authorized users.

## Introduction

Morphological traits of plants have evolved to adapt to the local environment. Bryophytes, the first land plants (Bowman et al. 2017), have been classified as mosses, hornworts, and liverworts. Unlike other terrestrial plants, bryophytes lack vasculature and roots, although they produce root-like structures. Since the surface of land was drier and more exposed to UV radiation than the interior of the sea, bryophytes needed to adapt to such harsh environmental conditions. This adaptation was fundamentally driven by genes and gene regulatory networks (GRNs), which represent the regulatory connections among genes. Because GRNs are not a physical entity and cannot be directly observed, rewiring within GRNs and/or appearance of new GRNs not associated with morphological changes can rarely be detected. Recent technical advances have allowed the precise survey of differences in gene expression on a genome-wide scale, thus enabling an in-depth analysis of GRNs. Transcription factors (TFs) are central to the constitution of a GRN. A TF regulates gene expression, and various genes regulated by multiple TFs are connected to one another, forming a GRN. TFs are classified into various types based on the protein structure, such as DNA-binding and protein−protein interacting regions. Genome sequences of plants suggest that, during evolution from charophyte green algae to bryophytes, certain TFs have remarkably increased in number (Catarino et al. 2016). The TF families that increased in size after terrestrialization are thought to be involved in the adaptation of plants to land environments. The basic helix-loop-helix (bHLH) family is one of the largest TF families in bryophytes (Bowman et al. 2017; Pires and Dolan 2010). Genes encoding bHLH proteins in the genome of *Marchantia polymorpha* L., a liverwort, are remarkably higher in number than those encoding other types of TFs such as MYB, suggesting the potential role of bHLH proteins in the development of new traits (Bowman et al. 2017; Pires and Dolan 2010). These traits are responsible for the evolution of new GRNs capable of protecting plants against environmental stresses on land. Although genome-wide approaches allow the analysis of GRNs, further studies are required for understanding how GRNs evolved and acquired new regulatory connections.

The evolution of GRNs is the most thoroughly studied in the seed plant *Arabidopsis thaliana* [see the review (Gupta and Tsiantis 2018)]. Various bHLH proteins involved in root hair formation in *A. thaliana* are also conserved in the moss, *Physcomitrella patens*. A common ancestor of the GRN of genes encoding bHLH proteins has been proposed to be involved in root hair formation; however, considerable hierarchical architectural changes throughout the evolution of plant vasculature (Pires et al. 2013; Tam et al. 2015). A GRN required for multiple biological events could help elucidate the acquisition of multiple regulatory networks by GRNs and the evolution of the GRN of genes encoding bHLH proteins. AtGL3, one of the bHLH proteins in *A. thaliana*, positively regulates anthocyanin accumulation and trichome formation but negatively regulates root hair formation by forming a complex with the R2R3-type MYB and WD40 repeat proteins, called the MBW complex. Notably, the MBW complex is conserved throughout the plant kingdom (Feller et al. 2011; Ishida et al. 2008; Lloyd et al. 2017; Xu et al. 2015). Depending on the biological process regulated by a GRN, AtGL3 forms the MBW complex with different MYB TFs. The GRN of *AtGL3* is a remarkable tool for elucidating the evolution of GRNs. According to Heim’s classification, AtGL3 belongs to the IIIf bHLH protein family (Heim et al. 2003). This family of proteins is thought to be involved in specific biological events, such as organ development and secondary metabolism, by forming MBW complexes; the specificity of GRNs depends on the partner MYB proteins (Lloyd et al. 2017; Xu et al. 2015). In *A. thaliana*, four bHLH genes are classified in the IIIf clade: *AtGL3, AtEGL3, AtTT8*, and *AtMYC1*. Although the precise biological functions of the proteins encoded by these genes are not yet understood, they are known to be involved in trichome development and flavonoid biosynthesis (Maes and Inze 2008; Morohashi 2009; Symonds et al. 2011). The IIIf bHLH proteins are involved in specific but varied biological events. Thus, it would be interesting to compare the GRN of IIIf bHLH genes between the ancient land plant, *M. polymorpha*, and the established seed plant, *A. thaliana*. Despite the conservation of the IIIf bHLH domain among seed plants, it has not yet been reported in ancient terrestrial liverworts, and the evolution of such a specific GRN of IIIf bHLH genes remains poorly understood.

The genome of *M. polymorpha* was recently sequenced (Bowman et al. 2017). In this study, we successfully isolated the gene encoding a IIIf bHLH TF from *M. polymorpha*, and named it *MpBHLH12*. Overexpression of *MpBHLH12* in *M. polymorpha* reduced the size and number of gemma cups. Transcriptomic analysis of *MpBHLH12* ectopic expression in *M. polymorpha* suggests that MpBHLH12 regulates genes with diverse functions, unlike the IIIf bHLH TFs in *A. thaliana*, which regulate genes involved in specific biological pathways. Our results bring a possibility that MpBHLH12 might acts as a transcriptional repressor rather than a transcriptional activator of gene expression. Nonetheless, *MpBHLH12* showed interaction with MYB proteins of the MBW complex of *A. thaliana*. Taken together, these findings suggest that a GRN of the IIIf bHLH TF in *A. thaliana* might have been evolved from the GRN of *MpBHLH12*, perhaps by gaining transcriptional activation activity.

## Materials and methods

### Plant materials

In this study, a male strain of *M. polymorpha*, Takaragaike-1 (Tak-1), and *A. thaliana* ecotypes Colombia (Col-0) and Landsberg (Ler) were used as wild strains.

### Plasmid construction

250 µL of super optimal broth with catabolite repression (SOC) medium (20 g L^−1^ Bacto Tryptone, 5 g L^−1^ Bacto Yeast Extract, 10 mM NaCl, 2.5 mM KCl, and 0.2 M glucose) was added to the mixture, and cells were cultured for recovery at 37 °C for 1 h. The cultured cells were transferred to Luria Bertani (LB) agar medium (10 g L^−1^ Bacto Tryptone, 5 g L^−1^ Bacto Yeast Extract, 10 g L^−1^ NaCl, and 15 g L^−1^ agar) containing appropriate antibiotics and incubated at 37 °C on a shaker at 220 rpm overnight. Colonies were then transferred to fresh LB agar medium containing appropriate antibiotics, and a master plate was produced. Subsequently, the transformed *E. coli* were cultured in liquid LB medium (10 g L^−1^ Bacto Tryptone, 5 g L^−1^ Bacto Yeast Extract, and 10 g L^−1^ NaCl) containing appropriate antibiotics, and plasmid DNA was isolated using the FastGene Plasmid Mini Kit (NIPPON GENE, Tokyo, Japan).

### pENTR_MpBHLH12

The coding sequence of *MpBHLH12* (*Mapoly0031s0161*), excluding the stop codon, was amplified by PCR using the KOD FX Neo enzyme solution (TOYOBO, Osaka, Japan). To reliably amplify the sequence of interest, nested PCRs were performed using cDNA template and different sequence-specific primers (Table S1). In the first PCR, primers HA_nest_Mapoly0031s0161_f and HA_nest_Mapoly0031s0161_r were used to amplify a sequence, including 100 bp both at the 5′ and 3′ ends of the target sequence. The nested PCR was performed by amplifying 1 µL of the first PCR solution as a nested PCR template to the desired sequence. For the nested PCR, HA_TOPO_Mapoly0031s0161_f and HA_TOPO_Mp_r were used. The final PCR product was purified by ethanol precipitation and cloned into pENTR/D-TOP (Thermo Fisher Scientific, Waltham, Massachusetts). After cloning, the plasmid was sequenced using the following four primers: HA_Mapoly_seq_p1, HA_Mapoly_seq_p2, HA_Mapoly_seq_p3, and HA_Mapoly_seq_p4. Only those plasmids that were cloned successfully and carried no mutations in the sequence, named pENTR_MpBHLH12, were used in further experiments.

### pMpGWB105_35S::Citrine:MpBHLH12, _35S::MpBHLH12:VP16:HSP, _35S::MpBHLH12:HSP, and _35S::GAL4DB:MpBHLH12

The pENTR_MpBHLH12 was used as an entry vector, and pMpGWB105, pDEST35S-VP16-HSP, pDEST35S-HSP, and pDEST430T were used as destination vectors for generating pMpGWB105_35S::Citrine:MpBHLH12, _35S::MpBHLH12:VP16:HSP, _35S::MpBHLH12:HSP, and _35S::GAL4DB:MpBHLH12 constructs. Cloning was performed using Gateway® LR Clonase ™ II Enzyme Mix (Thermo Fisher Scientific, Waltham, Massachusetts).

### LDOXp_FLUC_HSP

The promoter of leucoanthocyanidin dioxygenase (*LDOX*) was amplified by PCR using the primer pair YT_FLUC_LDOX_Fwd and YT_CLUC_FLUC_LDOX_Rev with PrimeSTAR® HS DNA polymerase (TAKARA Bio, Shiga, Japan). Primers were designed to produce an insert (proLDOX) containing homologous sequences with 20 bp overlaps to both ends of the vector. The PCR product was ligated with the digested pGL4.1_HSP using the NEBuilder® HiFi DNA Assembly Master Mix (NEB, Ipswich, Massachusetts), according to the manufacturer’s instructions.

### Plasmid transformation

For transformation, *Escherichia coli* competent cells stored at − 80 °C were thawed on ice. When the competent cells were thawed, a plasmid solution was added, and the mixture was allowed to stand on ice for 30 min. Next, the mixture was incubated at 42 °C for 1 min and then on ice for 2 min.

### Generation of transgenic *M. polymorpha*

Transformation of *M. polymorpha* was performed as described previously (Ishizaki et al. 2016). Briefly, *Agrobacterium tumefaciens* strain GV2260 was used for the transformation of Tak-1. Only *Agrobacterium* successfully transformed with pMpGWB105_35S::Citrine:MpBHLH12 formed colonies on the plate. Each colony was transferred to fresh LB agar medium with an appropriate antibiotic to prepare a master plate, and then cultured in liquid LB medium containing an appropriate antibiotic. The gemma cups of wild-type Tak-1 were cultured on 1/2 Gamborg’s B5 agar medium for 15 days. Next, slices cut from the growing point of thalli were prepared and cultured for 5 days in the same medium to induce the regeneration of gemma cups. Transformation generated 5−10 lines from each transgenic, and plasmid insertion was confirmed by genotyping. Individuals cultured for 1 week on 1/2 Gamborg’s B5 agar medium were used to isolate DNA for genotyping. Approximately five transgenic individuals were placed in a 2 mL tube containing DNA extraction beads with 100 µL of genotyping buffer (100 mM Tris–HCl [pH 9.5], 1 M KCl, and 10 mM EDTA). The tube was shaken at 3,000 rpm for 30 s, and the mixture was diluted five times with the genotyping buffer. PCR was performed using this mixture as the DNA template.

### Phylogenetic analysis

**Fig. 1:**
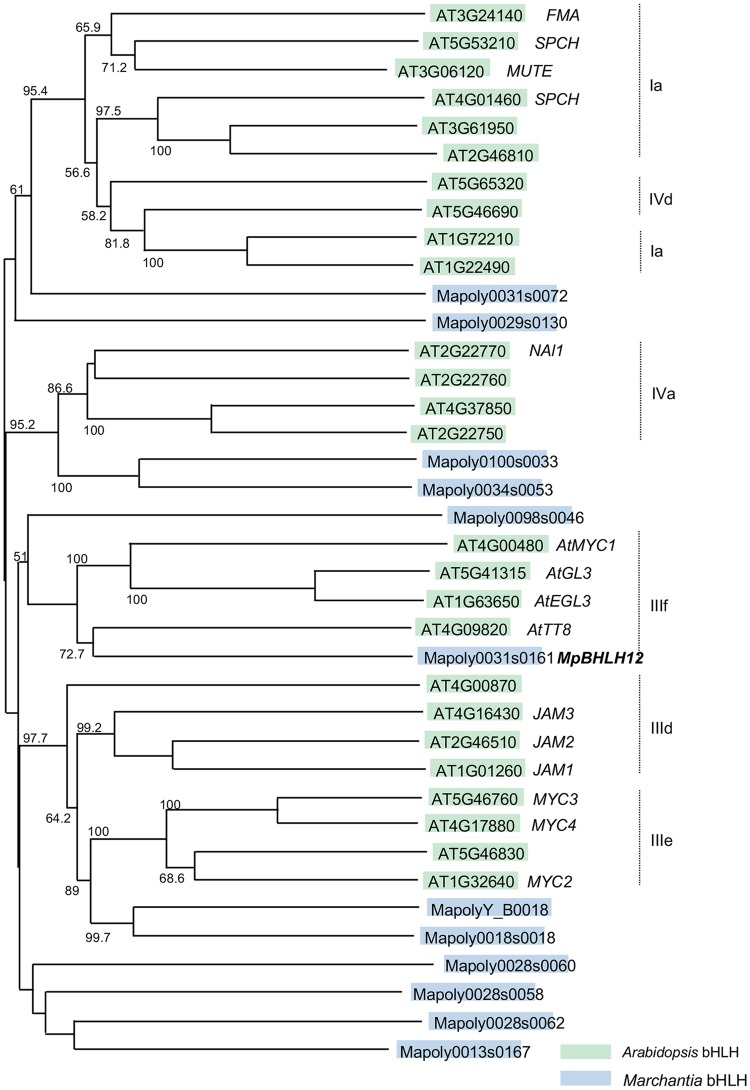
Phylogenic analysis of basic helix-loop-helix (bHLH) proteins in *Marchantia polymorpha* and *Arabidopsis thaliana*. The phylogenetic tree was drawn using the amino acid sequence of the bHLH transcription factors. Green and blue boxes indicate the bHLH proteins of *A. thaliana* and *M. polymorpha*, respectively. Gene names belonging to clade III are shown

A phylogenetic tree was constructed using the bootstrap neighbor-joining (N-J) method, with default parameters (1000 permutations), in Clustal X software (Thompson et al. 1997). The output was drawn using NJplot, with minor modifications (Perrière and Gouy 1996). We aligned putative bHLH protein sequences from Phytozome with AtGL3 amino acid sequence, followed by chosen genes encoding amino acid sequences close to IIIf clade. Deduced amino acid sequences of the following genes were used for alignment in Fig. 1: *AT3G24140, AT5G53210, AT3G06120, AT4G01460, AT3G61950, AT2G46810, AT5G65320, AT5G46690, AT1G72210, AT1G22490, AT2G22770, AT2G22760, AT4G37850, AT2G22750, AT4G00480, AT5G41315, AT1G63650, AT4G09820, AT4G00870, AT4G16430, AT2G46510, AT1G01260, AT5G46760, AT4G17880, AT5G46830, AT1G32640, Mapoly0031s0072, Mapoly0029s0130, Mapoly0100s0033, Mapoly0034s0053, Mapoly0098s0046, Mapoly0031s0161, MapolyY_B0018, Mapoly0018s0018, Mapoly0028s0060, Mapoly0028s0058, Mapoly0028s0062*, and *Mapoly0013s0167*.

### Measurement of gemma cup size

Wild-type Tak-1 and transgenic lines of *M. polymorpha* (MpBHLH12ox), in which MpBHLH12 was constitutively expressed, were cultured on 1/2 Gamborg’s B5 agar medium for 3 weeks. Subsequently, the area of 10 gemma cups from at least three independent thalli was measured using a digital microscope VHX-5000 (KEYENCE, Osaka, Japan).

### DNA extraction

*M. polymorpha* were cultured for 1 week in 1/2 Gamborg’s B5 liquid medium. Approximately 20 thalli were harvested, placed in 2 mL tubes containing beads, and frozen in liquid nitrogen. Frozen thalli were crushed with beads by shaking at 3,000 rpm for 30 s, and genomic DNA was extracted using the Wizard® Genomic DNA Purification Kit (Promega).

### RNA extraction and quantitative reverse-transcription PCR (qRT-PCR)

*M. polymorpha* were cultured on 1/2 Gamborg’s B5 agar medium for 4 weeks. RNA was extracted using the TRI Reagent (MOR Molecular Research Center), according to the manufacturer’s instructions. *M. polymorpha* were crushed using beads (3,000 rpm, 30 s). The extracted RNA was digested with DNaseI (Sigma–Aldrich, St. Louis, Missouri) and reverse transcribed using ReverTra Ace® qPCR RT Kit (TOYOBO, Osaka, Japan). The synthesized cDNA was amplified by qRT-PCR using the THUNDERBIRD® SYBR® qPCR Mix (TOYOBO, Osaka, Japan). Transcripts were quantified using the ^ΔΔ^Ct method. The *elongation factor-1α* gene (*EF1α; Mapoly0024s0116*) was used as a standard for data normalization (Table S1).

### RNA-Seq analysis and data mining

Wild-type Tak-1 and two independent transgenic *MpBHLH12* overexpresser lines (MpBHLH12ox#2 and #3) were cultured on 1/2 Gamborg’s B5 agar medium for 3 weeks. Nine pieces (approximately 5 × 5 mm) of thalli were harvested from each MpBHLH12ox line and placed in a 2 mL tube containing beads. These samples were frozen with liquid nitrogen and crushed by shaking the tube at 3,000 rpm for 30 s. Total RNA was extracted from the crushed thalli using Nucleo Spin® RNA Plant Kit (MACHEREY–NAGEL, Düren, Germany), according to the manufacturer’s instructions, and sent to Dr. Kimura (Department of Life and Resources Environment, Kyoto Sangyo University School of Life Sciences), where sequencing was performed by NextSeq500 with NextSeq 500 High Output Kit v2 (Illumina, San Diego, USA).

Fastq sequence data were mapped onto the reference genome of *M. polymorpha* (JGI_3.1.fasta) using Bowtie2 (Langmead and Salzberg 2012), and the number of fragments was determined using HTseq (Anders et al. 2015). Next, the number of fragments was counted with EdgeR (Robinson et al. 2010), and the magnitude of the fluctuation in expression was calculated as log_2_ (fold-change). Genes with significant differences in expression between wild-type Tak-1 and transgenic lines (MpBHLH12ox#2 and #3) were considered differentially expressed. Using EdgeR, multiplicity correction was applied by the Benjamini-Hochberg method on the *p* values, to control the false discovery rate shown as FDR. Gene Ontology (GO) analysis was performed using the Plant Transcriptional Regulatory Map (Plant RegMap; Center for Bioinformatics Peking University, http://plantregmap.cbi.pu.edu.cn/go.php). In this tool, annotations of the Gene UniProt Ontology Annotation Database (European Molecular Biology Laboratory, https://www.ebi.ac.uk/GOA) and InterProScan (European Molecular Biology Laboratory, https://www.ebi.ac.uk /interpro/interproscan.html) were used. For each GO term, Fisher’s exact test was used to determine the statistical significance of GO enrichoment. RNA-Seq data from this study were deposited in Gene Expression Omnibus (accession number: GSE119305).

### Measurement of anthocyanin content

Wild-type Tak-1 and two independent transgenic MpBHLH12ox lines were cultured for 10 days in 1/2 Gamborg’s B5 liquid medium lacking phosphorus. Each *M. polymorpha* sample was divided into three tubes, frozen in liquid nitrogen, and stored at − 80 °C. The frozen *M. polymorpha* samples were lyophilized for 24 h and weighed. Approximately 40 µL of MeFA buffer (MeOH:H_2_O:Formic acid = 10:47:3) per mg dry weight of the plant was added to the sample, and the suspension was incubated overnight at room temperature in the dark. The following day, absorbance of 1.5 µL of the suspension was measured (OD = 532 and 657). An arbitrary unit of anthocyanin analog was calculated using the following equation (Mancinelli 1990):

$$Amount\,of\,anthocyanin\,~analog\,=\,O{D_{523}} - \left( {0.25~ \times ~O{D_{657}}} \right)$$

### Transient reporter assays

Plants of *A. thaliana* ecotype Col-0 were grown on a plate for 3 weeks under short day conditions (light: 8 h, dark: 16 h) using 1/2 MS gellan gum medium. After 3 weeks, plants were transplanted in soil (vermiculite:soil = 1:1) at a spacing of approximately 7 cm and grown for 2 months under short day conditions. Plants were watered with a 1,000-fold dilution of HyPONeX.

Approximately 20 mL of enzyme solution, 0.15 g Cellulase “Onozuka” R10 (Yakulto, Tokyo, Japan), 0.035−0.04 g Mecerozyme R10 (Yakulto, Tokyo, Japan), 14 µL β-mercaptoethanol, and digestion buffer (400 mM mannitol, 20 mM KCl, 10 mM CaCl_2_, 20 mM MES [pH 5.7]), were contained. The enzyme solution was degassed twice for eliminating bubbles. To isolate protoplasts, eight pieces (1.0−1.5 cm) of *A. thaliana* leaves were attached to a mending tape, and the epidermis was peeled off, exposing the mesophyll cells. Approximately 24 pieces of epidermis-stripped leaves were soaked in the enzyme solution (20 mL) in a 50 mL tube and incubated at 22 °C on a shaker at 60 rpm for 1-1.5 h. The suspension was then filtered through a 70 µm cell strainer, and the filtrate was collected in a 50 mL tube. The tube used for enzymatic reaction and the used tape pieces were washed with 20 mL of W5 buffer (150 mM NaCl, 125 mM CaCl_2_, 5 mM KCl, and 2 mM MES [pH 5.7]). The suspension was then filtered through a 70 µm cell strainer into the tube containing previously filtered cells. The sample was subjected to centrifugation at 100×*g* for 10 min, and the supernatant was discarded. The precipitate was gently resuspended in 20 mL of W5 buffer by pipetting and centrifuged at 100×*g* for 10 min. This washing step was repeated three times. Finally, cells were washed with 20 mL of MMg solution (400 mM mannitol, 15 mM MgCl_2_ and 4 mM MES [pH 5.7]). After centrifugation at 100×*g*, protoplasts were suspended in 2.5 mL of MMg solution, and the number of protoplasts was measured using a hemocytometer.

For transient transformation experiments using *A. thaliana* leaf protoplasts, plasmids were resuspended in sterile ultrapure water. First, 10 µL of plasmid solution, containing reporter plasmid (500 µg), effector plasmid (100 µg), and reference plasmid (pRL; 10 µg), was dispensed into 2 mL round bottom tubes. *Note*: using flat bottom tubes or 1.5 mL tubes markedly reduces the transformation efficiency. Next, 70 µL of the protoplast suspension was added to each tube using the same pipette tip, followed by the addition of 90 µL of polyethylene glycol (PEG) solution (40% [w/v] PEG 4000 [Fluka, ‎Morris Plains, New Jersey], 0.2 M Mannitol, and 0.1 M CaCl_2_). Protoplasts were gently vortexed at 900−1,700 rpm and incubated at room temperature for 10 min. Subsequently, W5 buffer was added vigorously at 400 µL, and the cells were suspended by convection. After centrifugation at 100×*g* for 5 min, 400 µL of the supernatant was removed by placing the tip of the pipette near the curved bottom of the tube. Protoplasts were washed with W5 buffer repeatedly, until clean clumps were obtained. After transformation, protoplasts were incubated at 22 °C for at least 12 h.

Protoplasts settled at the bottom of the tube were gently resuspended by vortexing. Subsequently, 40 µL of 5 × cell lysate was dispensed, and lysates were vortexed at 900−1,700 rpm for 15 s. Next, 16 µL of each lysate was transferred to two tubes. The Pikka Gene Dual Assay Kit (Toyo Ink, Tokyo, Japan) was used for the dual luciferase assay. In the first tube, the substrate of firefly luciferase (FLuc) was added, and luciferase activity was measured 20 times s^−1^ for 20 s. The substrate for *Renilla* luciferase (RLuc) was added into the second tube, and luciferase activity was measured as described above. The average value of 20 s measurements was calculated and normalized relative to the number of cells. The average value obtained using the FLuc substrate was divided by that obtained using the RLuc substrate. This value of luciferase activity was compared with the negative control, which was represented by a plasmid encoding a vesicle-associated membrane protein (VAMP) added as an effector.

## Results

### Isolation of the *MpBHLH12* gene encoding IIIf bHLH TF

To gain insight into the evolution of GRNs, we aimed at characterizing the GRN of genes encoding IIIf bHLH proteins in *M. polymorpha*. First, we searched for IIIf bHLH homologs of AtGL3 (At5g41315) in *M. polymorpha* using the deduced amino acid sequence database of Phytozome v12.1. A deduced amino acid sequence of a single gene (*Mapoly0031s0161*) showed the highest similarity with that of AtGL3; this gene is named *MpBHLH12* in MarpolBase. Phylogenetic analysis of bHLH amino acid sequences in *M. polymorpha* and *A. thaliana* indicated that MpBHLH12 belongs to the IIIf clade together with AtGL3, AtTT8, AtMYC1, and AtEGL3 (Fig. 1). Genes encoding IIIf bHLH proteins often contain seven to eight exons. Notably, MpBHLH12 harbors nine exons, and the positions of exon–intron junctions closely corresponded to those of AtGL3, confirming that MpBHLH12 belongs to the IIIf clade.

The IIIf bHLH proteins show specific structural features such as the presence of a bHLH domain in the middle of the protein and an ACT domain at the C-terminus (Feller et al. 2011). Sequence similarities in these domains between *A. thaliana* bHLH proteins and *M. polymorpha* MpBHLH12 are summarized in Table 1. Sequence similarities between the ACT domains among the *A. thaliana* IIIf clade bHLH proteins ranged from 23.6 to 93.1%, and those between the ACT domains of MpBHLH12 and *A. thaliana* IIIf bHLH proteins ranged from 22.2 to 27.8%. Additionally, sequence similarities in the bHLH domain among *A. thaliana* IIIf bHLH proteins ranged from 38.0% 88.0%, and those between the bHLH domain of MpBHLH12 and *A. thaliana* IIIf bHLH proteins ranged from 34.0 to 68.0%, suggesting that the bHLH domain of MpBHLH12 was also highly conserved. Among the Arabidopsis IIIf bHLH proteins, AtTT8 showed the highest similarity with MpBHLH12 in the bHLH domain, whereas AtGL3 showed the highest similarity in the ACT domain.

**Table 1:** Percent identity between the amino acid sequence of IIIf bHLH proteins of *Arabidopsis thaliana* and the MpBHLH12 protein of *Marchantia polymorpha*

	MpBHLH12	AtGL3	AtEGL3	AtTT8	AtMYC1
ACT domain (%)					
MpBHLH12		27.8	22.2	26.4	27.8
AtGL3			93.1	25.0	41.7
AtEGL3				25.0	40.3
AtTT8					23.6
AtMYC1					
bHLH domain (%)					
MpBHLH12		52.0	52.0	68.0	34.0
AtGL3			88.0	54.0	42.0
AtEGL3				54.0	42.0
AtTT8					38.0
AtMYC1					

### *MpBHLH12* suppresses gemma cup developments

**Fig. 2:**
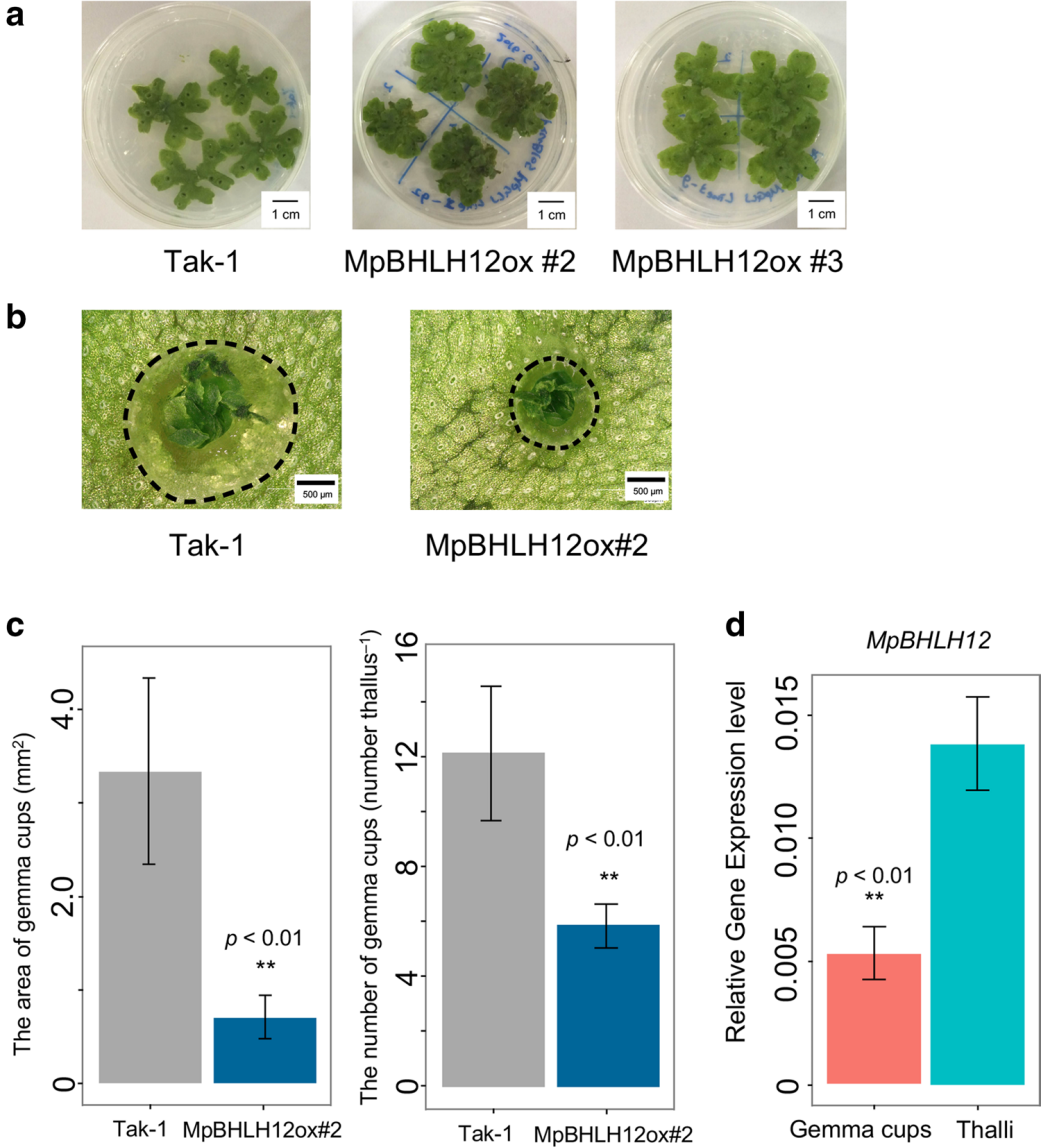
Morphological features of *MpBHLH12* overexpressor (MpBHLH12ox) lines. **a** Phenotypes of the thallus of wild-type Tak-1 (left), MpBHLH12ox#2 (middle), and MpBHLH12ox#3 (right) grown on 1/2 Gamborg’s B5 agar medium for 3 weeks (scale bar = 1 cm). **b** Images of gemma cups of 3-week-old wild-type Tak-1 (left) and MpBHLH12ox#2 (right) captured under a digital microscope (scale bar = 500 µm). **c** Area of gemma cups of 3-week-old Tak-1 and MpBHLH12ox#2. The area of gemma cups was approximately fourfold lower in MpBHLH12ox#2 than in wild-type Tak-1 (**p < 0.01; *n* = 10). **d** Quantitative real-time PCR (qRT-PCR) analysis of *MpBHLH12* expression in the gemma cups and thalli of MpBHLH12ox#2 (**p < 0.01; *n* = 4)

To identify a GRN involving *MpBHLH12* in *M. polymorpha*, we constitutively overexpressed a translational fusion of Citrine, a yellow fluorescent protein, and MpBHLH12 in *M. polymorpha*. Two independent MpBHLH12ox lines were generated: MpBHLH12ox#2 and MpBHLH12ox#3. We confirmed that *MpBHLH12* was constitutively expressed in these transgenic lines, as the Citrine foci were localized to the nucleus (Fig. S1), supporting our assumption that MpBHLH12 functions as a TF. Thallus sizes of MpBHLH12ox lines and Tak-1 did not show significant difference (data not shown), and we did not detect visible phenotypic differences between wild-type Tak-1 and MpBHLH12ox lines, except for the size and number of gemma cups (Fig. 2a), both of which were reduced in the transgenic lines MpBHLH12ox#2 and MpBHLH12ox#3 (Fig. 2b). The size of gemma cups was fourfold smaller in MpBHLH12ox lines than in wild-type Tak-1 (Fig. 2c). Additionally, MpBHLH12ox lines produced approximately half as many gemma cups as wild-type Tak-1 (Fig. 2c). Moreover, in 4-week-old wild-type Tak-1, the expression of MpBHLH12 in gemma cups was significantly lower than that in thalli (Fig. 2d).

### *MpBHLH12* does not induce anthocyanin accumulation

**Fig. 3:**
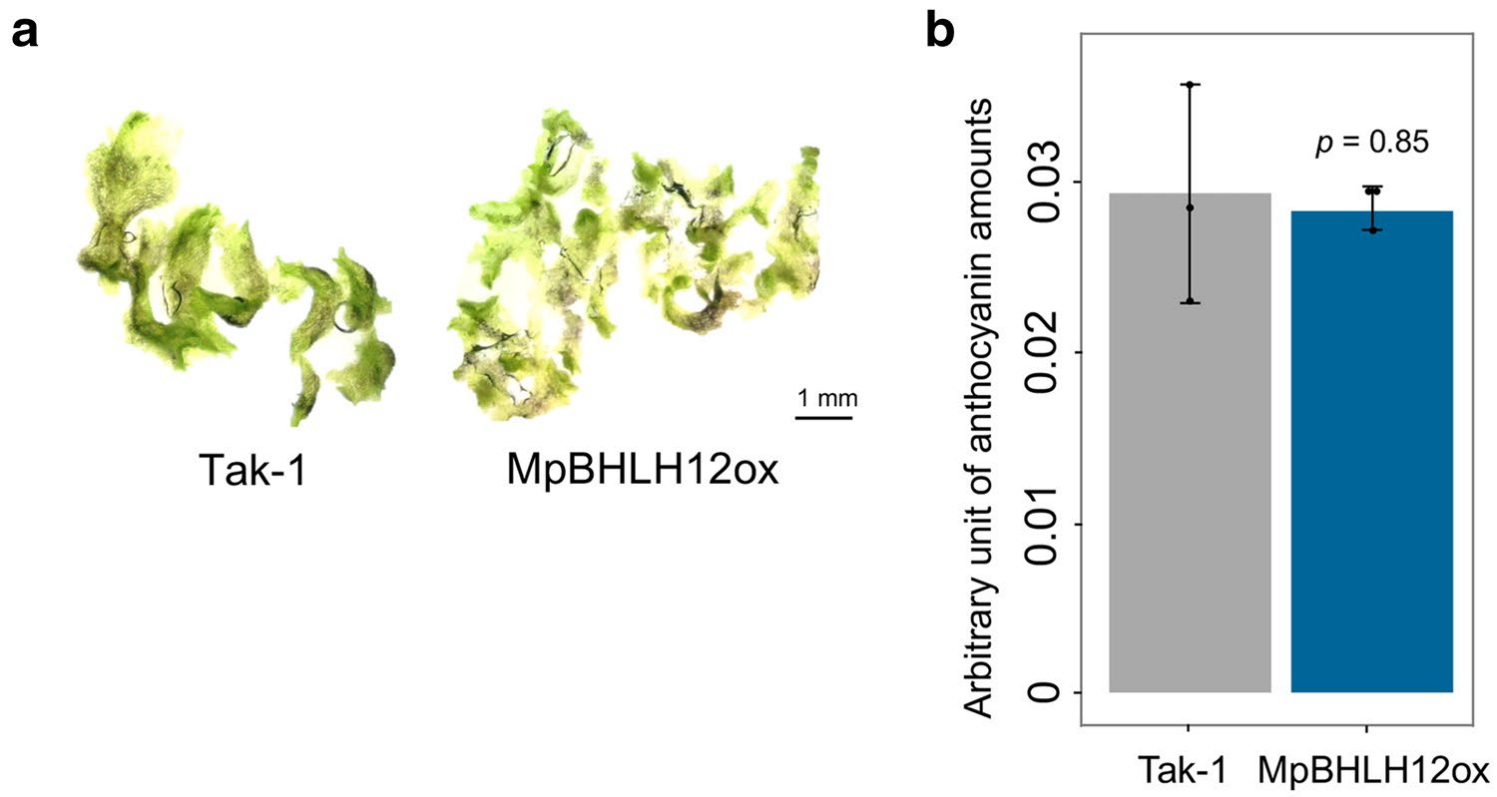
Measurement of anthocyanin content in wild-type Tak-1 and MpBHLH12ox lines. Tak-1 and MpBHLH12ox#8 were grown on 1/2 Gamborg’s B5 culture medium lacking phosphorus for 1 week. **a** Images of thalli of Tak-1 (left) and MpBHLH12ox#8 (right) captured under a digital microscope (scale bar = 1 mm). In both Tak-1 and MpBHLH12ox#8, a part of the thallus showed brown discoloration. **b** Quantification of anthocyanin content in the thalli of 1-week-old Tak-1 and MpBHLH12ox#8 (**p < 0.01; *n* = 3). Anthocyanin content was measured in an arbitrary unit. MpBHLH12ox#2 and #3 were shown similar phenotypes as well

Since IIIf bHLH proteins in *A. thaliana* regulate not only epidermal organ differentiation but also anthocyanin accumulation, we investigated the accumulation of anthocyanins in MpBHLH12ox lines based on the appearance of dark pigmentation. Under normal growth conditions, neither wild-type Tak-1 nor MpBHLH12ox lines showed dark pigmentation. Nonetheless, all plants exhibited a small amount of dark brown pigmentation in thalli when grown in a nutrient depleted medium known to induce anthocyanin production in *M. polymorpha* (Albert et al. 2018); however, the amount of anthocyanin-related metabolites extracted from wild-type Tak-1 and MpBHLH12ox lines showed no significant differences (Fig. 3). These data suggest that MpBHLH12 does not induce the accumulation of anthocyanins in *M. polymorpha* tissues.

### RNA-Seq analysis

**Fig. 4:**
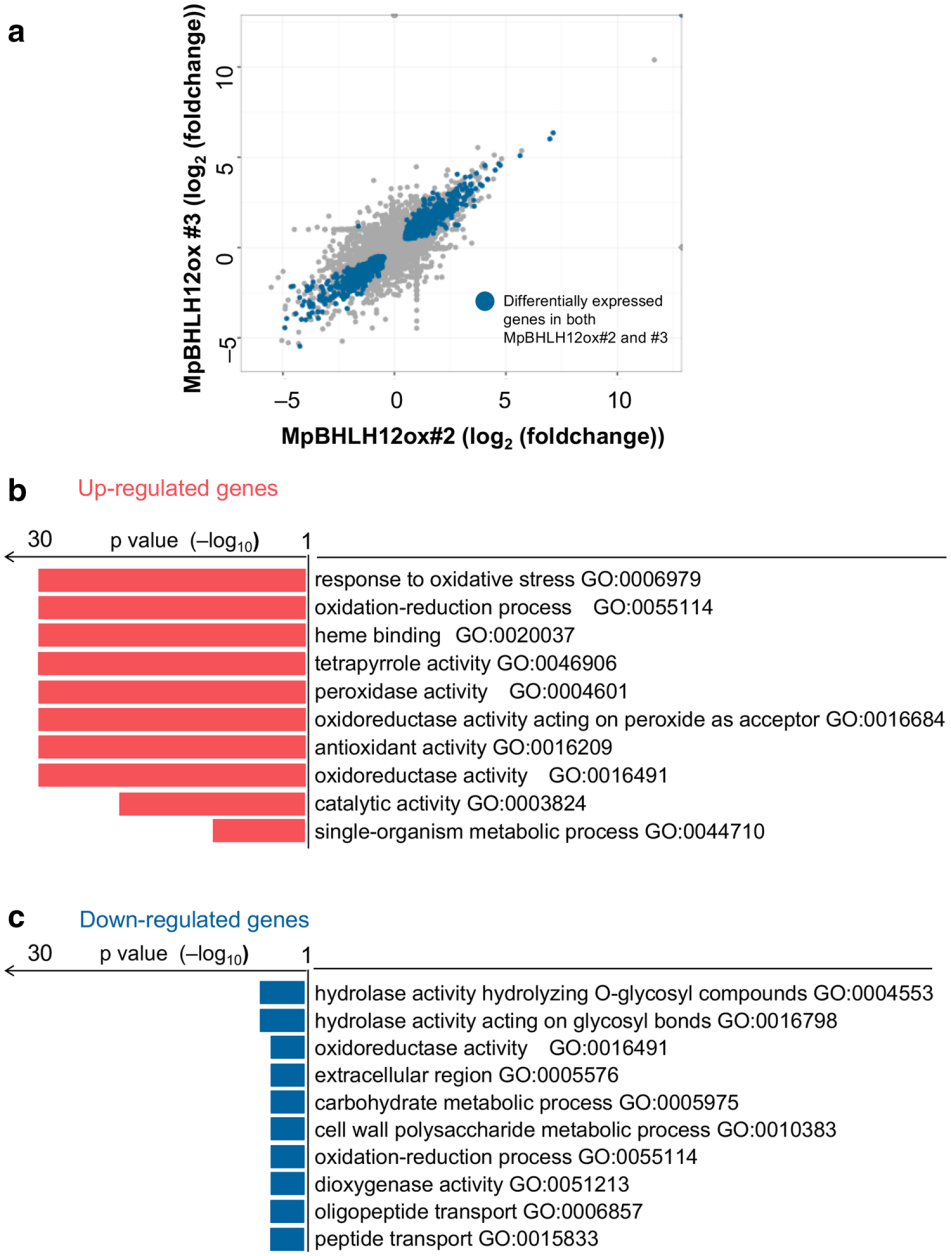
Analysis of the transcriptome of MpBHLH12ox lines. **a** Scatter plot of MpBHLH12ox#2 and #3. X- and Y-axes indicate log_2_ (fold-change) in gene expression in MpBHLH12ox#2 and #3, respectively, relative to that in Tak-1 A total of 1,205 genes were differentially expressed in both overexpressor lines. **b, c** Gene ontology (GO) analyses of the up-regulated (**b**) and down-regulated (**c**) genes. All significantly enriched GO terms (*p* < 0.01) are shown

To further investigate the GRN of *MpBHLH12*, we performed RNA-Seq analysis of both MpBHLH12ox lines and wild-type Tak-1. The overall expression of genes in MpBHLH12ox#2 and #3 was significantly correlated (Pearson’s correlation coefficient [*r*] = 0.89; *p* < 2.2 × 10^−16^), suggesting that our RNA-Seq results were derived from the effects of ectopic expression of *MpBHLH12* and did not depend on the transgenic lines used (Fig. 4a). A total of 1,205 genes were differentially expressed between the Tak-1 and transgenic lines (FDR < 0.05; Table S2). Of these 1,205 genes, 647 were up-regulated and 558 were down-regulated in MpBHLH12ox lines compared with the wild type. Moreover, GO enrichment analysis showed that, among the up-regulated genes, those involved in the response to oxidation and oxidative stress were significantly enriched (Fig. 4b). Among the down-regulated genes, some of the GO terms related to oxidation were markedly enriched, although none of the GO terms were significantly enriched (Fig. 4c).

**Fig. 5:**
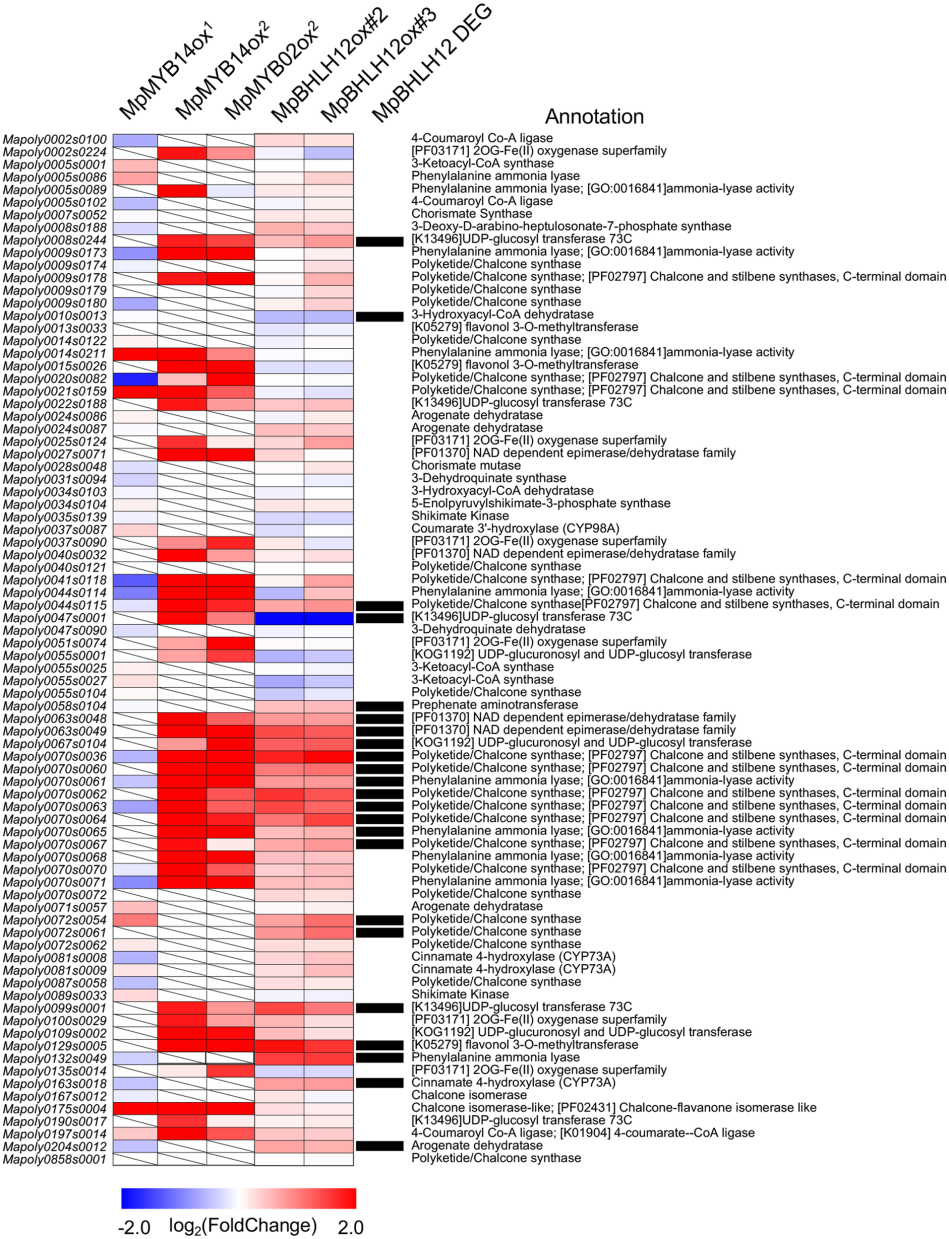
Heat map showing the comparison of shikimate and flavonoid pathway genes among MpBHLH12ox lines (this study) and transgenic *M. polymorpha* lines overexpressing *MpMYB14* and *MpMYB02* (Albert et al. 2017; Kubo et al. 2018). Color indicates log_2_ (fold-change) in gene expression in overexpressor lines compared with that in wild-type Tak-1; degree of difference is shown from blue to red. Differentially expressed genes (DEGs) identified in MpBHLH12ox lines are shown in a black box. Genes with no expression data available are indicated with diagonal lines

Although we did not observe anthocyanin accumulation in MpBHLH12ox#2 and MpBHLH12ox#3, we explored the genes involved in flavonoid biosynthetic pathways in *M. polymorpha*. Recently, *MpMYB14* and *MpMYB02* have been reported to regulate the accumulation of anthocyanidins (such as riccionidin) and phenolics, respectively, specific to *M. polymorpha* (such as marchantin) (Albert et al. 2018; Kubo et al. 2018). While MpMYB02 showed no significant difference in expression between the transgenic lines and the wild type, MpMYB14 expression was significantly up-regulated in both MpBHLH12ox lines compared with the wild type (average log_2_ [fold-change] = 0.77; FDR < 0.05). Since *MpMYB14* regulates genes involved in the shikimate and phenylpropanoid pathways, we compared the expression of 1,205 DEGs with those of 82 genes involved in the shikimate and phenylpropanoid pathway (Albert et al. 2018; Kubo et al. 2018). Our results showed that 23 of 82 genes genes were differentially expressed between the Tak-1 and transgenic lines, and this difference in expression was statistically significant (*p* < 0.001; Fisher’s exact test; Fig. 5, Table S3). Previously, Kubo et al. and Albert et al. generated transgenic *M. polymorpha* lines constitutively overexpressing *MpMYB14* and performed RNA-Seq analysis (Albert et al. 2018; Kubo et al. 2018). We assessed the fold-change in *MpMYB14* expression in MpBHLH12ox lines. Correlation of fold-changes in the expression of 23 genes between MpBHLH12ox and MpMYB14ox was modestly positive (*r* = 0.28; *p* = 0.09), which is consistent with the up-regulation of *MpMYB14* in MpBHLH12ox. Although MpBHLH12ox lines failed to induce anthocyanin production, GRNs of MpBHLH12 and MpMYB14 may overlap.

### Functional validation of MpBHLH12

**Fig. 6:**
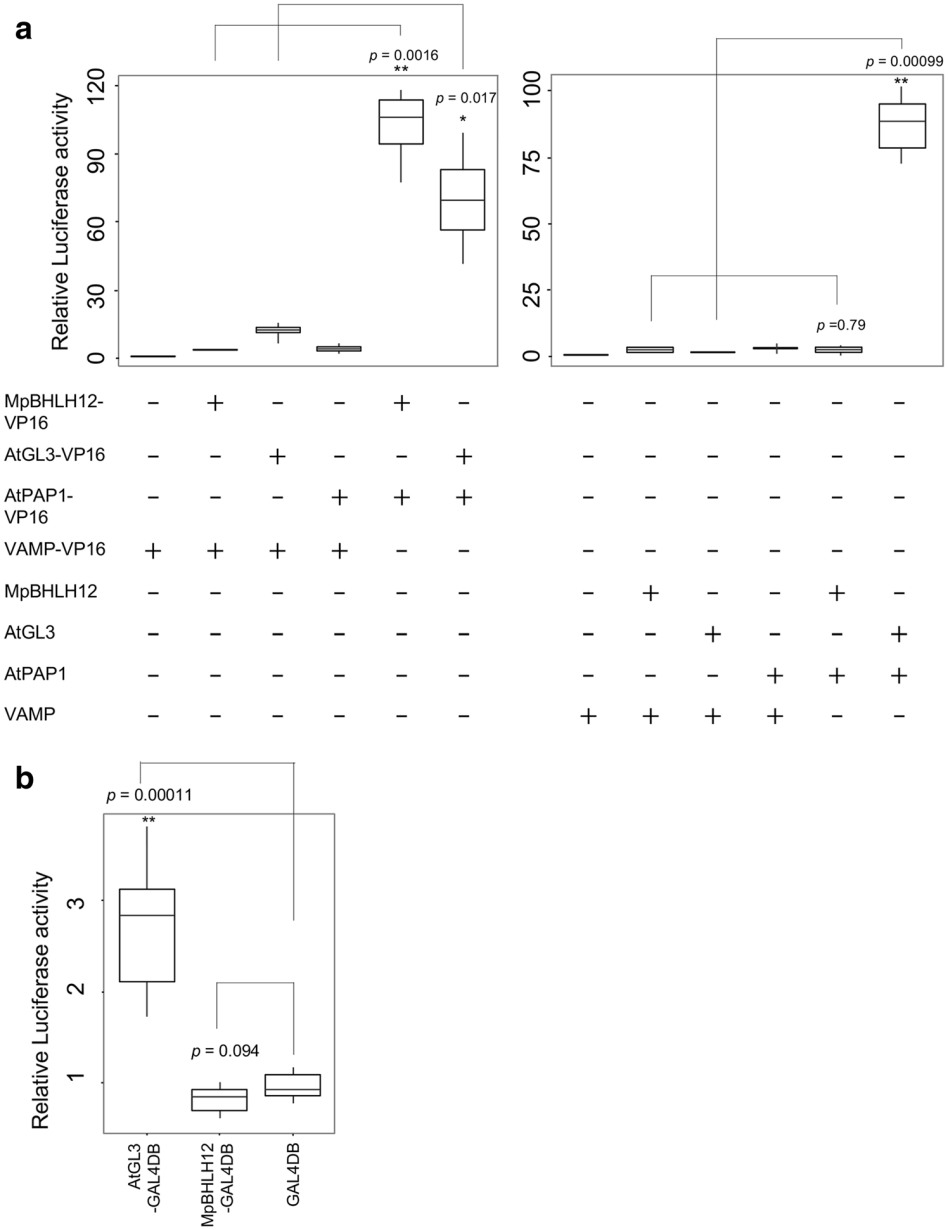
Transient reporter assays performed using *Arabidopsis* protoplasts. **a** Plasmid expressing luciferase (LUC) under the control of the *AtLDOX* promoter was used as a reporter. The transcription factor (TF) used in each effector plasmid is shown as (+) under the boxplots. Plasmid encoding a vesicle-associated membrane protein (VAMP) was used as a negative control. Statistically significant data are indicated with asterisks (*p < 0.05; **p < 0.01; *n* = 4). **b** Plasmid expressing LUC under the control of a GAL4-binding sequence (UAS) was used as a reporter (*n* = 8). GAL4DB is bound to UAS. Activity of the protein added as an effector was measured

Our results suggest that MpBHLH12 belongs to the IIIf clade of bHLH proteins, which are involved in epidermal differentiation and anthocyanin accumulation, and likely participate in the development of gemma cups but do not regulate anthocyanin accumulation. These data raise the question whether the molecular function of MpBHLH12 is similar to that of IIIf bHLH proteins in *A. thaliana*. We tested whether MpBHLH12 functions as a TF in *A. thaliana* protoplasts, as a heterologous system. Since the activation of target genes requires only MYB and bHLH proteins in *A. thaliana*, we used AtGL3 and AtPAP1 as controls. Promoter of the *AtLDOX* gene, which encodes an enzyme of the anthocyanin biosynthetic pathway in *A. thaliana* (Appelhagen et al. 2011), was used to drive the expression of the reporter gene. Protoplasts prepared from *A. thaliana* leaves were used for the transient reporter assays. The results of these assays showed that MpBHLH12 failed to activate the *AtLDOX* promoter alone and in the presence of AtPAP1, whereas AtGL3 activated the *AtLDOX* promoter in the presence of AtPAP1 (Fig. 6a). These results suggest at least two possibilities: (1) MpBHLH12 exhibits transactivation activity but does not associate with AtPAP1, or (2) MpBHLH12 associates with AtPAP1 but does not exhibit transactivation activity. To identify the most plausible hypothesis, we generated a CaMV35S::*MpBHLH12:VP16* construct, which was reinforced to show strong transactivation activity with MpBHLH12. Strong activation of the *AtLDOX* promoter was observed when MpBHLH12:VP16 was co-expressed with AtPAP1:VP16 in the protoplasts, suggesting that MpBHLH12 associates with AtPAP1 but does not show transactivation activity. Furthermore, because RNA-Seq suggested the possibility that MpBHLH12 could not function as a activator, we measured the repressive activity of MpBHLH12 using the GAL4 system. MpBHLH12 was fused to the GAL4 DNA-binding domain (GAL4DB), which binds to the upstream activation sequence (UAS) fused to a reporter gene. Fusion of AtGL3 with the GAL4DB (AtGL3:GAL4DB) strongly activated the UAS promoter. By contrast, the MpBHLH12:GAL4DB fusion modestly repressed the UAS (Fig. 6b). These results suggest that, unlike AtGL3, MpBHLH12 does not exhibit transactivation activity but shows repressive activity. Overall, our results suggest that MpBHLH12 belongs to the IIIf clade of bHLH proteins, and the transcriptional activity of MpBHLH12 is different from that of the *A. thaliana* IIIf bHLH protein, AtGL3.

## Discussion

In this study, we isolated the gene encoding IIIf bHLH protein, *MpBHLH12*, from *M. polymorpha* and characterized the GRN of this gene. In *A. thaliana*, IIIf bHLH proteins regulate multiple biological events such as the biosynthesis of flavonoids (particularly anthocyanins) and formation of trichomes and root hairs. According to Fig. 1, MpBHLH12 is the closest homolog to the Arabidopsis IIIf bHLH proteins; clades IIId and IIIe are distant from MpBHLH12. Therefore, we speculated that MpBHLH12 would have similar functions to those of IIIf bHLH proteins in higher plants.

In liverwort, gemma cups develop from the epidermal cells of thalli. Since the IIIf bHLH proteins in *A. thaliana* participate in epidermal cell development, the reduction in the size and number of gemma cups in MpBHLH12ox lines could be a characteristic of the IIIf bHLH proteins. However, a comparison between the morphologies of *A. thaliana* and *M. polymorpha* is not straightforward and requires further investigation.

It has been recently shown that *M. polymorpha* accumulates flavones and anthocyanidins (riccionidin A), and *MpMYB14* increases the amount of these flavonoids (Albert et al. 2018; Kubo et al. 2018). In this study, we found that MpBHLH12 activated *MpMYB14* expression. Moreover, some of the genes regulated by MpMYB14 were also affected in MpBHLH12ox lines. These findings suggest that the GRN of *MpBHLH12* is partially similar to that of *MpMYB14*. However, MpBHLH12ox lines did not show dark pigmentation, although *M. polymorpha* constitutively expressing *MpMYB14* showed red pigmentation. This could be because MpMYB14 functions via an alternative pathway, leading to anthocyanidin accumulation, rather than a common pathway with MpBHLH12. However, this possibility has not yet been confirmed, and metabolomics analysis may provide further clues. Furthermore, we found differences in the expression of 82 genes predicted to be involved in shikimate and flavonoid pathways. *Mapoly0072s0061* (predicted to encode chalcone synthase) was significantly up-regulated in MpBHLH12ox lines compared with Tak-1. Another interesting gene obtained from the comparison between MpBHLH12ox and *MpMYB14* ectopic expression lines is *Mapoly0047s0001*, which putatively encodes UDP-glucosyl transferase. *Mapoly0047s0001* was the only gene significantly down-regulated in MpBHLH12ox lines; however, this gene was significantly up-regulated in *MpMYB14* ectopic expression lines. According to a recent publication by Clayton et al. (2018), UVB irradiation induces flavonoids and does not induce *MpBHLH12* expression. Moreover, the expression of chalcone isomerase and chalcone isomerase-like genes induced by UVB is not affected in MpBHLH12ox lines, suggesting that the GRN of *MpBHLH12* is distinct from that of flavonoid biosynthesis genes regulated by MpMYB14. Gene expression profiles of MpMYB14ox in two groups by Albert et al. (2018), and Kubo et al. (2018), were slightly different (Fig. 5). This could be due to their growth condition and/or different background strain, Tak-1 in Kubo group and our experiments, and Sey-1 and Aud-2 in Albert group of MpMYB14ox which might have affected the GRN involving MpMYB14; therefore, direct comparison of MpMYB14ox and MpBHLH12ox under identical environmental condition might be required. At present, we do not have sufficient information to predict the relationship of GRNs of MpBHLH12 and MpMYB14; transcriptomics using inducible MpBHLH12 might help elucidate the dynamics of metabolic pathways.

We predicted that MpBHLH12 was an activator because conserved amino acids in the IIIf bHLH proteins responsible for transactivation were also conserved in MpBHLH12 (Fig. S2). However, MpBHLH12 did not activate the *AtLDOX* gene promoter, despite its association with AtPAP1. We propose that MpBHLH12 functions in gene repression rather than activation. It is possible that the IIIf bHLH proteins harbor an unknown conserved region that affects transcriptional activity. Further studies on IIIf bHLH proteins in other terrestrial plants are needed for elucidating the mechanism of GRNs of genes encoding IIIf bHLH proteins.

## Electronic supplementary material

Below is the link to the electronic supplementary material.

Supplementary material 1 (PDF 348 KB)

Supplementary material 2 (XLSX 2282 KB)

Supplementary material 3 (XLSX 17 KB)
