# Supplementary material for: Genome-wide analysis of MpBHLH12, a IIIf basic helix-loop-helix transcription factor of Marchantia polymorpha
Source: J Plant Res. 2019 Mar 6;132(2):197–209. doi: 10.1007/s10265-019-01095-w (PMC7196945; doi:10.1007/s10265-019-01095-w)
Supplement: Supplementary file 1 — Supplementary material 1 (PDF 348 KB) [file 10265_2019_1095_MOESM1_ESM.pdf]

Electronic supplementary materials

Title:
Genome-wide analysis of MpBHLH12, a IIIf basic helix-loop-helix transcription factor of *Marchantia polymorpha*

Authors:
Haruka Arai, Kazuya Yanagiura, Yuko Toyama, Kengo Morohashi

Department of Applied Biological Science, Faculty of Science and Technology, Tokyo University of Science, 2641 Yamazaki, Noda, Chiba 278-8510, Japan

Journal:
Journal of Plant Research

Corresponding author:
Kengo Morohashi

E-mail:
morohashi.1@rs.noda.tus.ac.jp

Content:
Figs. S1–S2
Tables S1

Figure S1.

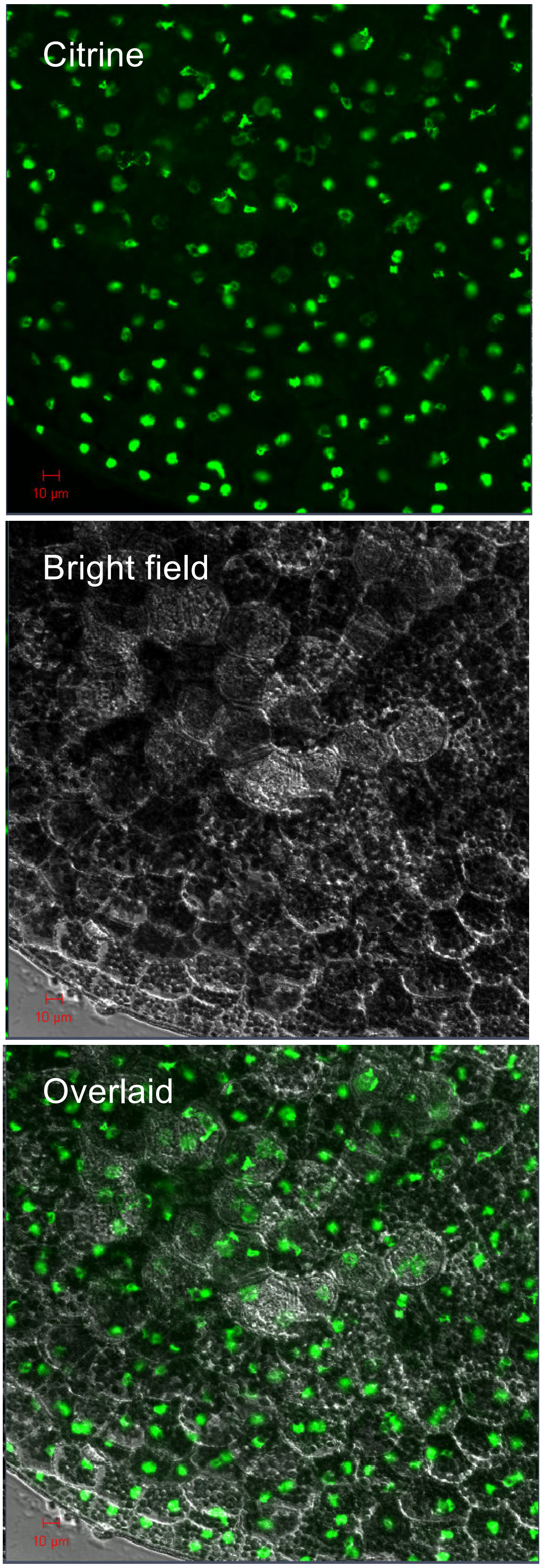

Fig S1. Citrine expression in MpBHLH12ox#2. MpBHLH12:Citrine fluorescence was observed as foci in a nucleus. Top, middle and bottom panels show citrine, blight field and overlaid pictures, respectively. Scale bars = 10 µm

Figure S2.

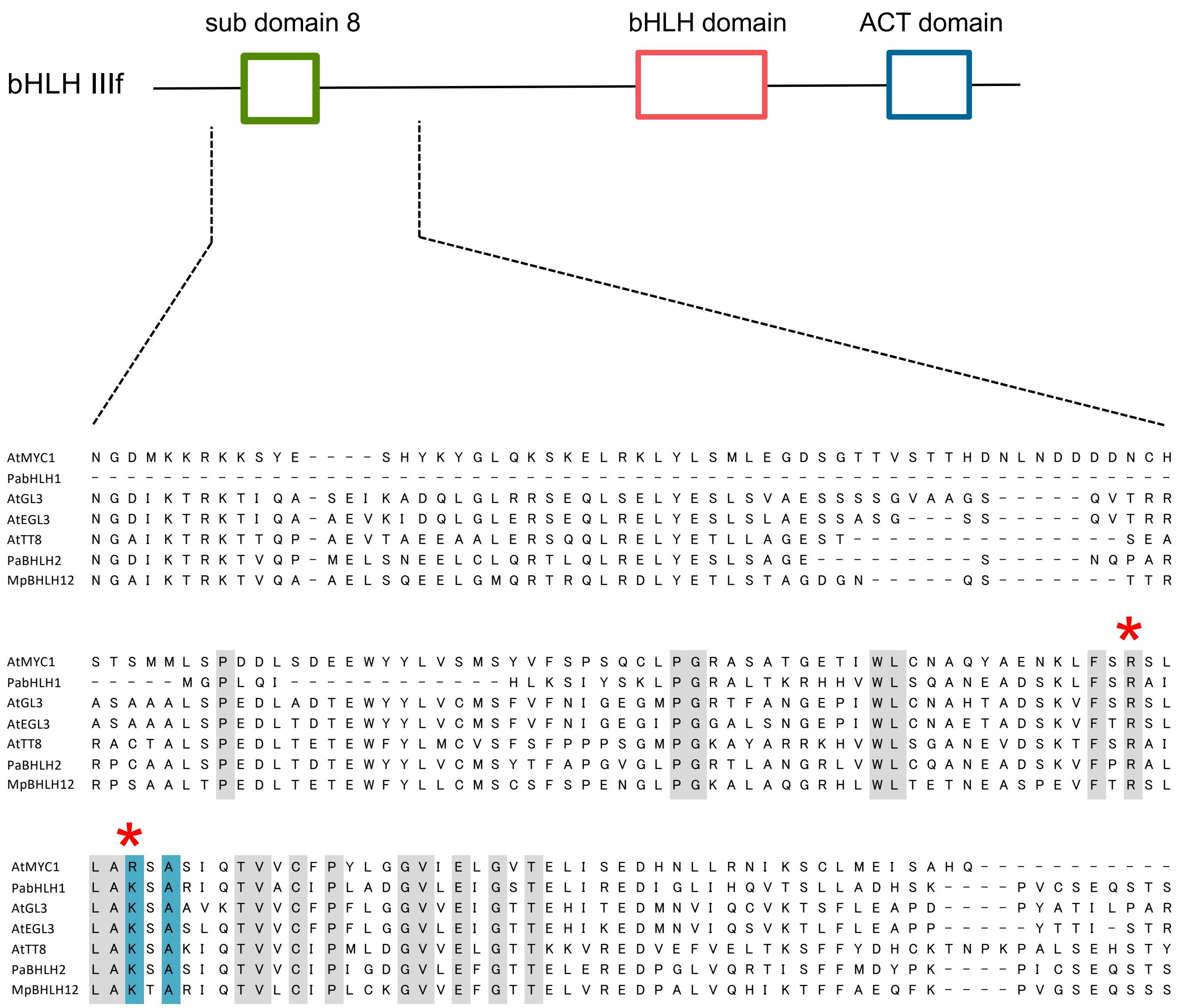

Fig S2. Amino acid alignment of IIIf-clade bHLHs from *A. thaliana, P. patens,* and *M. polymorpha*. The portion shown in gray is an amino acid that was common among all bHLHs. Light blue indicates an amino acid involved in transcriptional activation. Red asterisk indicates an amino acid known to affect association with R2R3 MYB.

Table S1.

Table S1. Primer list used in this manuscript

Primer name	Sequence
HA_nest_Mapoly0031s0161_f	5'-AAACGATTCGGCCTGCAGTAATCG-3'
HA_nest_Mapoly0031s0161_r	5'-GCTTGCATTGCAGCTCAGTGTAG-3'
HA_TOPO_Mapoly0031s0161_f	5'-CACCATGGCTGGAGCGGGCAAAAGAAAT-3'
HA_TOPO_Mp_r	5'-TCTGGAGCCCATCCCAGCAGCGG-3'
HA_qrt_Mapoly_seq_p1	5'-CGAGCTCAGTCAAGAGGAAC-3'
HA_qrt_Mapoly_seq_p2	5'-CAACAGCCAGGACGAAGTGG-3'
HA_qrt_Mapoly_seq_p3	5'-CTTCGTCGACTGGAGAATGAC-3'
HA_qrt_Mapoly_seq_p4	5'-CGTGAATGTCAGCTTCGACG-3'
HA_qrt_Mp0031s0161_2_f	5'-CGTGCTCATTCTCGCCGG-3'
HA_qrt_Mp0031s0161_2_r	5'-GGGCTGGCCTCATTGGTC-3'
HA_qrt_MpEF1_F	5'-AAGCCGTCGAAAAGAAGGAG-3'
HA_qrt_MpEF1_R	5'-TTCAGGATCGTCCGTTATCC-3'
HA_qrt_Mapoly0028s0058_F	5'-GGTCCATCCCGCCGCTCATGA-3'
HA_qrt_Mapoly0028s0058_R	5'-GAGTTGAACAGTCGCTCGAC-3'
HA_qrt_Mapoly0046s0038_2F	5'-CGAGGCGGCCATGCAGATTC-3'
HA_qrt_Mapoly0046s0038_2R	5'-GATTGGACTGCGTCGAGGTG-3'
HA_qrt_Mp0001s0298_F	5'-CTCCGGCTGGACTTCGCG-3'
HA_qrt_Mp0001s0298_R	5'-GATCTCTTTCCACTGGCC-3'
HA_qrt_Mapoly0041s0025_F	5'-CCACCTCCGAGCTCCAAGCTC-3'
HA_qrt_Mapoly0041s0025_R	5'-CGGCACTGGCGGATTCTTCG-3'
HA_qrt_Mapoly0166s0010_F	5'-CGAGTTTCACTCCGCGCCTC-3'
HA_qrt_Mapoly0166s0010_R	5'-CTCCTCTGTGTAGCTCGCTG-3'
KM qrt newPDF2-F	5'-TCCGAGATCACATGTTCCAAACTC-3'
KM qrt newPDF2-R	5'-CCGTATCATGTTCTCCACAACCG-3'
YT_qrt_GL3_F	5'-ACCGTCAATTGCAAGCACAAG-3'
YT_qrt_GL3_R	5'-GCAACCCTTTGAAGTGCTTCTTTG-3'
YT_qrt_LDOX_F	5'-TTGGCTAACAACGCGAGTGGAC-3'
YT_qrt_LDOX_R	5'-GCGTACTCACTCGTTGCTTCTATG-3'
